# Luetic Meningitis of the Anterio Cranial Fossa with Uncinate Gyrus Syndrome Simulating Neoplasm

**Published:** 1911-09

**Authors:** Tom A. Williams

**Affiliations:** M. B. C. M. (Edin.); Washington, D. C.; Corresp. Memb. Paris Neurolog. et Psychol Societies, etc., Neurologist to Epiphany Dispensary


### LUETIC MENINGITIS OF THE ANTERIO CRANIAL FOSSA WITH UNCINATE GYRUS SYNDROME SIMULATING NEOPLASM.

By Tom A. Williams, M. B. C. M. (Edin.), Washington, D. C.

*Corresp. Memb. Paris Neurolog. et Psychol Societies , etc., Neurologist to Epiphany Dispensary.*

A woman from West Virginia, aged 41, was referred by Dr. Wilmer, of Washington. She had complained of pains in the eyeball and feelings of distress in the vertical and lateral muscles there, and she is annoyed at having to turn her whole head when she changes the direction of her look.

*Previous History.* Although she had no previous visual difficulties, the muscles of the eyes have always troubled her, and ten years ago similar pains and muscae volitantes lead her to consult Dr. Wilmer and receive relief. She had had malaria until ten years ago, when she left Washington. Eleven months after her marriage a miscarriage occurred, and after this a severe oophoritis, which was cured by electrical treatment. She had several other early miscarriages, and a child which died at once after a prolonged labor.

A uterine discharge persists, in spite of curettage and cervectomy, but she has no discomfort, weakness or pain since the operation, and otherwise feels in good health as a rule, but at times she becomes exhausted and nervous, sometimes to the point of tears: from his she is often relieved by a drive or a walk. These attacks might last for a week, but did not cause insomnia. The last three months she has been somewhat depressed, and in the mornings feels as though a great stone lay on her chest.

*Present Condition.* There is dull frontal headache and an occasional throbbing above the left orbit. No dizziness, nausea rarely, although regurgitation often occurs. There often occurs a sensation in the nostrils as of an odor, which permeates everything she eats, but is like a static electric machine or ozone tube. It may last only a few minutes or some hours, and it always occurs suddenly and quite independently of her thoughts.

*The Reflexes.* Patella lively and equal. Achilles left greater than right. Triceps over active, left greater than right. Plantar, the great toe is immobile, the movement of lesser toes is very slight, especially to left.

Abdominal is also absent, but vol tional contractions make it hard to examine. The response is at least irregular and delayed.

*Sensibility* was normal to wool, pin, deep pain, cold, heat, attitude and vibration, except that over the dorsum of the right foot the tuning fork is felt as a burning, and she believes that it is still vibrating while stationary. As this mistake was not committed upon the arms, a slight defect of sensibility in the lower limbs may be inferred. There was no loss of smell or taste. There was no hemianopia.

*Motility.* No weakness, except perhaps in the facial movements, nor paralysis; no ataxia. The eye movements were complete. The diadokokinesis, however, was less quickly performed with the left arm. There was no dysergia on mounting a chair or leaning back.

The pulse beat 120 during examination. The urine was variable in quantity, but no abnormal constituents were found. She seemed soporific, but declared herself to feel nervous and excited. Although she declared herself to be of a nervous family, no stigmata of hypersuggestibility were found, nor had she become subject to phobias, anxiety, manias or mannerisms. The following report of the case was accordingly made:

"I have examined your patient, Mrs. S. The only significant signs that I found were a slight optic papillary pallor, an inequality of the Achilles reflexes, the left being more active than the right; a similar inequality in the triceps and radial, a diminution of the plantar reflexes, especially in the left; an occasional misinterpretation of sensations of deep pressure upon the lower limbs, it sometimes being called heat; a relative diadokokinesis in the movements of the left wrist, and an apparent soporific state during examination. She declared on the contrary that she felt excited, and her pulse frequency of 120 per minute bears this out. There are no objective signs to enlighten me about her parosmic symptom, and it might possibly be elucidated by a rhinologist.

The symptoms (headache, depression and regurgitation without cause) and signs, now present concord with a neoplasm of very slow growth pressing upon the uncinate gyrus in the neighborhood of the optic nerve, chiasm or tract. The relative exaggeration of the reflexes of the left side being due to pressure from a distance on the motor projection fibres, and the sudden olfactory paraesthesia being due to the vascular modifications which oscillate to such a degree within the cranium. I do not believe that pressure on the olfactory tract or bulb would occur without giving rise to some loss of smell, which is not present. The symptoms, however, are not enough for a positive diagnosis as yet; and I recommend that she be kept under observation, and seen again at the end of six months.

It is possible that the Wasserman reaction might throw light on the etiology and guide us in treatment.

This appeared to show that the last supposition was correct, as Dr. Wilmer informed me a year ago that the symptoms had cleared up after potassium iodide had been given.

In a forthcoming synthesis, this case contributes to the differential diagnosis where pathological changes in the brain simulate the symptoms caused by neoplasm there.

1758 K. St.

---

### SURGICAL SUGGESTIONS.

Severe neuralgic pain over the bridge of the nose indicates pressure on the anterior ethmoidal nerve probably due to a high deviation of the nasal septum.

A peritonsillar abscess as a rule is more painful than serious. But one should not forget that patients have died of suffocation and that erosion of a vessel may take place in the wall of the cavity and cause death.

A severe sore feeling in the throat is frequently complained of by nervous individuals. Close inspection will show numerous fine white spots surrounded by a red areola—herpes.

A small erosin of the trachea may give rise to a distressing hemoptysis which differs from a hemorrhage from the lungs in that there are no lung symptoms, no loss of weight or constitutional symptoms and in that the bleeding occurs in small lumps of clotted blood.

Pressure from a mediastinal tumor or enlarged tubercular glands will give rise to an irritative condition of the throat which can in no way be relieved by local measures.

If a patient prepared for ureterolithotomyy has a sudden surcease or an exacerbation of pain—and even without these if the stone is quite small—have a final skiagraphic exposure just before operating. If the stone has slipped into the bladder it is better for both patient and surgeon to discover this by the x-ray than by the knife.—American Journal of Surgery.

